# Modulation of immune-responses by DSF/Cu enhances the anti-tumor effects of DTX for metastasis breast cancer

**DOI:** 10.7150/jca.89120

**Published:** 2024-01-21

**Authors:** Jingtian Guo, Yuxiao Ma, Tiejun Tang, Zhengying Bian, Qianwen Li, Lei Tang, Zhuolan Li, Mengyuan Li, Liming Wang, Aizhong Zeng, Shihao Huang, Wei Guo

**Affiliations:** Jiangsu Key Laboratory of Druggability of Biopharmaceuticals, State Key Laboratory of Natural Medicines, School of Life Science and Technology, China Pharmaceutical University, Nanjing 211198, PR China.

**Keywords:** metastasis breast cancer, immunogenic cell death, disulfiram

## Abstract

Metastasis has been one of the most important causes of death from breast cancer, and chemotherapy remains the major option for metastatic breast cancer. However, drug resistance and higher toxicity from chemotherapy have been an obstacle for clinical practice, and the combination of chemotherapy with immunotherapy has emerged as a promising treatment strategy. Here, we describe a therapy based on the combination of disulfiram (DSF) and Cu^2+^ with widely used cytotoxic docetaxel (DTX). DSF/Cu-induced immunogenic cell death promoted the release of type I interferon and human monocyte-induced dendritic cell maturation, which established a foundation for the combination with chemotherapy. Consequently, the combination of DSF/Cu and DTX resulted in significantly more potent anti-tumor effects in 4T1-bearing mice than in single therapy. The present study has shed new light on combining DSF/Cu-induced immune responses with traditional chemotherapeutic agents to achieve greater benefits for patients with metastasis.

## Introduction

With 2.261 million confirmed cases in 2020, breast cancer ranks first among all cancers, which poses a serious threat to women's health^1^. Metastasis is the leading cause of death in breast cancer, with about 20-30% of patients developing metastases and about 90% of cancer-related deaths attributed to metastases. The 5-year survival is over 80% for patients without metastases, compared to 25% for those with metastases. Chemotherapy remains the mainstay in treating metastases breast cancer, and the preferred drugs include anthracyclines, taxanes, anti-metabolites and microtubule inhibitors. However, due to drug resistance and toxicity, combination therapy and immunotherapy has gained considerable interest from researchers, which is gradually becoming the current trend for the treatment of refractory breast cancer.

Through changing the immunosuppressive microenvironment, immunotherapy transforms "cold tumors" into "hot tumors" to activate the host's immune response and induce long-term anti-tumor responses. Combining immunotherapy with chemotherapy promises cumulative effects and inhibits further tumor progression^2^. G. Kroemer's group found that some chemotherapeutic drugs not only have direct cytotoxic effects on cancer cells but also activate the anti-tumor response of CD8^+^ T cells in mice, which defined as immune primary cell death (ICD)^3^. ICDs are recognized as a novel immunomodulation for cancer therapy that stimulates immune responses to dead cell antigens^4^. Once cancer cells undergo ICD, they release damage-associated molecular patterns (DAMPs), such as adenosine triphosphate, high mobility group box 1 (HMGB1), and IFN-β into the extracellular environment and transfer calreticulin (CALR) to the cell surface^4^. These blockers promote the phagocytosis, processing, and presentation of antigens from dendritic cells (DCs) to T lymphocytes, and further enhance the long-term antitumor effects^5^. In other words, dying cancer cells act as endogenous vaccines to stimulate the host's systemic antitumor immunology response. Enhancing the immunogenicity of breast cancer to improve the responsiveness to chemotherapy is expected to become an entry point for the treatment of metastases breast cancer.

Disulfiram (DSF) is approved by the U.S. Food and Drug Administration (FDA) for the treatment of alcohol addiction^6^. In 1976, E F Lewison found that the spontaneous regression of breast cancer patients was related to taking DSF, which first confirmed the anti-tumor effect of DSF^7^. The anti-tumor activity of DSF was proved in various cancers including prostate cancer and pancreatic ductal adenocarcinoma, and the specific mechanism was also identified in subsequent studies^8^,^9^,^10^. The study found that DSF exerts its anti-tumor effect in a Cu^2+^-dependent manner, which is related to the significantly higher copper levels in the serum and tumor tissue of patients than in healthy people. Therefore, DSF/Cu exhibits a natural targeting for tumor tissue^11^,^12^. DSF/Cu can inhibit the stemness of breast cancer stem cells (BCSC), suggesting potential value for breast cancer treatment^13^. Our previous study showed that DSF/Cu exhibits antitumor effects in pancreatic ductal adenocarcinoma and breast cancer partly dependent on DSF-induced autophagy-dependent apoptosis^14^. As a marketed medicine, DSF has a well-established safety profile. Regarding the safety of combination of DSF and Cu in clinical treatments, hepatobiliary disorders (NCT03363659, NCT03034135) and fatigue (NCT03034135) were reported as the major adverse events. Thus, whether DSF/Cu could act as an ICD inducer to enhance the immunogenicity of tumor cells for the improvement of therapeutic outcomes by traditional chemotherapy has drawn our attention to investigate.

In this study, we chose the common chemotherapy drug docetaxel (DTX), which has been proven not to induce ICD, in combination with DSF/Cu. The contribution of DSF/Cu to ICD and the efficacy of combination therapy were verified *in vitro* and *in vivo*. Thus, our work aims to adopt more effective tumor combination therapy strategies, including enhance the immunogenicity of breast cancer cells and provide new solutions for the treatment of metastatic breast cancer.

## Materials and methods

### Cell lines and mice

Mouse breast cancer cells 4T1 were purchased from American Type Culture Bank (ATCC) and were supplemented with 10% fetal bovine serum (FBS; Gibco, US), 100 U/mL streptomycin sulfate and 100 g/mL in RPMI1640 medium (Gibco, US) mL ampicillin sodium culture. MDA-MB-231 was purchased from Duke Comprehensive Cancer Center, USA, MCF-7 was purchased from Cell Bank of Chinese Academy of Sciences, and cultured in 1640 medium (Gibco, US) supplemented with 10% fetal bovine serum (FBS; Gibco, US), and Add double-antibody. All cells were cultured in a 37°C, 5% CO2 cell incubator.

Female BALB/c mice (6-8 weeks old) were purchased from Beijing Life River Laboratory Animal Technology Co., Ltd. (China). Animals were acclimated to specific pathogen-free conditions for one week before all experiments. All animal experiments were performed in accordance with the protocol of the Institutional Animal Care and Use Committee of China Pharmaceutical University.

### Reagents and antibodies

Tetraethylthiourea (DSF) was purchased from R&D (USA), and CuCl2-2H2O was purchased from Sigma (USA). DSF and CuCl2 were dissolved in DMSO and Milli-Q water, respectively. A stock solution of DSF (10 mM) was aliquoted and stored at 20°C for up to 1 year and freshly diluted with cell culture medium (in vitro assays) or PBS (in vivo assays) prior to use.

Fluorochrome-conjugated anti-mouse CD3e, anti-mouse CD8, anti-mouse CD25, anti-mouse Foxp3, anti-mouse CD8a, anti-mouse CD4 were purchased from BD Bioscience. Anti-human HLA, anti-human CD80, anti-human CD86, and anti-human CD11c were purchased from BioLegend, UK.

Western blotting analysis was performed using antibodies provided by Cell Signal Technology (US): HSP70 Rabbit mAb、HSP90 Rabbit mAb, STING Rabbit mAb, Phospho-STING(Ser366) Rabbit mAb, TBK1/NAK Rabbit mAb, Phospho-TBK1/NAK (Ser172) Rabbit mAb, IRF-3 XP® Rabbit mAb, Phospho-IRF-3(Ser396) Rabbit mAb. β-actin Rabbit mAb was purchased from Protetech (China).

Antibodies for immunohistochemical (IHC) analysis, including anti-mouse CD8a, anti-mouse F4/80, and anti-mouse LY6G (all from Cell Signaling Technology).

### Enzyme-linked immunosorbent assay (ELISA)

6×10^5^ cells were seeded in a six-well plate, and the adherent cells were treated with the indicated concentration of DSF/Cu. After 24h, the cells and supernatant were separated by centrifugation. The content of HMGB1 in the supernatant was detected by double antibody sandwich ELISA with the standard as a reference. 4T1 cells were used Mouse HMGB1 ELISA Kit (BG-MUS11178; Novatein Biosciences, USA), and MDA-MB-231 cells were used Human HMGB1 ELISA Kit (ARG81185; Arigobio, Taiwan, China). By detecting the OD value of 450nm, the concentration of experimental Wells was calculated by making standard curves.

### RNA extraction and real-time quantitative PCR

Total RNA was obtained from DSF/Cu-treated breast cancer cells and DC cells using EasyPure@RNA Kit (Transgen, China). 1 μg of total RNA was reverse transcribed into cDNA using 5×HiScript III qRT SuperMix kit (Vazyme, China). Performed on an Applied Biosystems real-time quantitative PCR instrument using SYBR Green Master Mix (Vazyme, China) under standard thermal cycler conditions according to the manufacturer's protocol. According to the relative quantitative (ΔΔCT) comparison method, compared with the control group, each group of primers was set as a negative control, and the positive control was β-actin. The sequences of qPCR primer pairs are shown in Table 1.

### Western blot analysis

After pretreatment with DSF/Cu or co-culture with pretreated tumor cells for 48 h, breast cancer cells or DCs were collected, and then protease and phosphatase inhibitors (TERMO, US) were added in 1 × RIPA buffer (TERMO, US). ) cracked. Add 5x loading buffer containing 5% β-mercaptoethanol to the protein sample and boil for 5~10min. Equal amounts of total proteins were separated by 10% SDS-PAGE and electrotransferred to polyvinylidene fluoride (PVDF) membranes. Specific proteins were detected with mouse, rabbit primary antibodies and horseradish peroxidase (HRP)-conjugated secondary antibodies. The secondary antibody bound to the PVDF membrane was reacted with ECL detection reagent (Thermo, USA). Results were normalized to the internal control β-actin.

### Human monocyte-derived dendritic cell differentiation

Peripheral blood mononuclear cells (PBMC) were extracted from the peripheral blood of healthy donors with human lymphocyte separation medium, all individuals provided informed consent to participate in this study. PBMCs were diluted to 3 x 10^6^ cells/mL in RIPA 1640 medium (Gibco, US) containing 800 U/mL recombinant human GM-CSF (R&D Systems, USA) and 500 U/mL IL-4 (R&D Systems, US). PBMCs were incubated for 12 hours to obtain adherent monocytes, and immature DCs were obtained by co-culture of GM-CSF and IL-4 for 4 days. On day 6, untreated tumor cells (negative control), pretreated dead tumor cells and 100 U/mL human TNF-α (positive control) were added to stimulate DC maturation. On the 8th day, mature DCs were taken for detection.

### Animal studies

Female Balb/c mice were subcutaneously inoculated with 4×10^5^ 4T1 cells (100 μL) on the right side and randomly divided into 4 groups according to tumor volume on the 3rd day after injection. Mice were injected intratumorally with 1.5 μM DSF and 1 μM Cu (100 μL) or 20 mg/kg DTX as a single or in combination. All mice were sacrificed on the 30th day after modeling, and tumors and lungs were fixed with 10% formalin and embedded in paraffin. Tumors were analyzed by immunohistochemistry (IHC), lung sections were stained with hematoxylin-eosin (H&E) for metastasis analysis, and spleens were removed for flow cytometry analysis. All animal studies were approved by the Institutional Animal Care and Use Committee.

### Immunohistochemistry (IHC)

Collected tumor issues were fixed by 4% paraformaldehyde and then embedded in paraffin. The paraffin-embedded tissues were cut into sections as thin as 4 to 5 μm with a microtome. After de-paraffinization, the samples were processed to block the endogenous target and nonspecific sites via 3% H2O2 and BSA respectively. The samples were incubated with the primary antibodies (CD8, F4/80 and Ly6G) overnight in 4℃ and then bound with the secondary antibody. After washing, the sections were viewed by light microscopy.

### Flow cytometry

2 × 10^6^ tumor cells were cultured in 6-well plates, and tumor cells were collected after DSF/Cu treatment for 24 h. Cells were washed twice with cold PBS and stained with specific antibodies for 30 min to detect cell surface antigens. Intracellular staining was fixed with BD Cytofix/Cytoperm (BD Biosciences, US) and permeabilized for 20 min at 4°C, followed by intracellular staining. Apoptosis levels were detected by Annexin V/7-AAD Apoptosis Detection Kit (640922, BioLegend, UK), and Alexa Fluor® 488-conjugated calreticulin rabbit mAb (Cell Signal Technology, US) was used to detect mouse and human Levels of cell surface calreticulin.

To assess immune cell subsets in tumor-bearing mice, spleens were collected at sacrifice. The ground spleen was filtered through a 70 μM cell strainer to obtain a single cell suspension, and the single cells of the spleen were subjected to CD3^+^, CD4^+^, CD8a^+^, CD25^+^, Foxp3^+^ cell staining analysis.

Flow cytometry was performed using Accuri C6 (BD Biosciences, USA) and data were analyzed using Flowjo V10 software (TreeStar, US).

### Statistics

Graphpad Prism 8.0 was used for statistical analysis of the data, Flowjo V10 software was used to process the flow experiment data, and Image J was used for grayscale scanning of Western Blot development bands and IHC analysis. Values are presented as mean ± standard error (x ± SEM). Student's t-test was used to analyze the significant differences between two groups, and One-way ANOVA was used to analyze the significant differences between multiple groups. p < 0.05 indicated that the difference was statistically significant.

## Results

### DSF/Cu induced tumor cell death and DAMPs release

**Figure 1:**
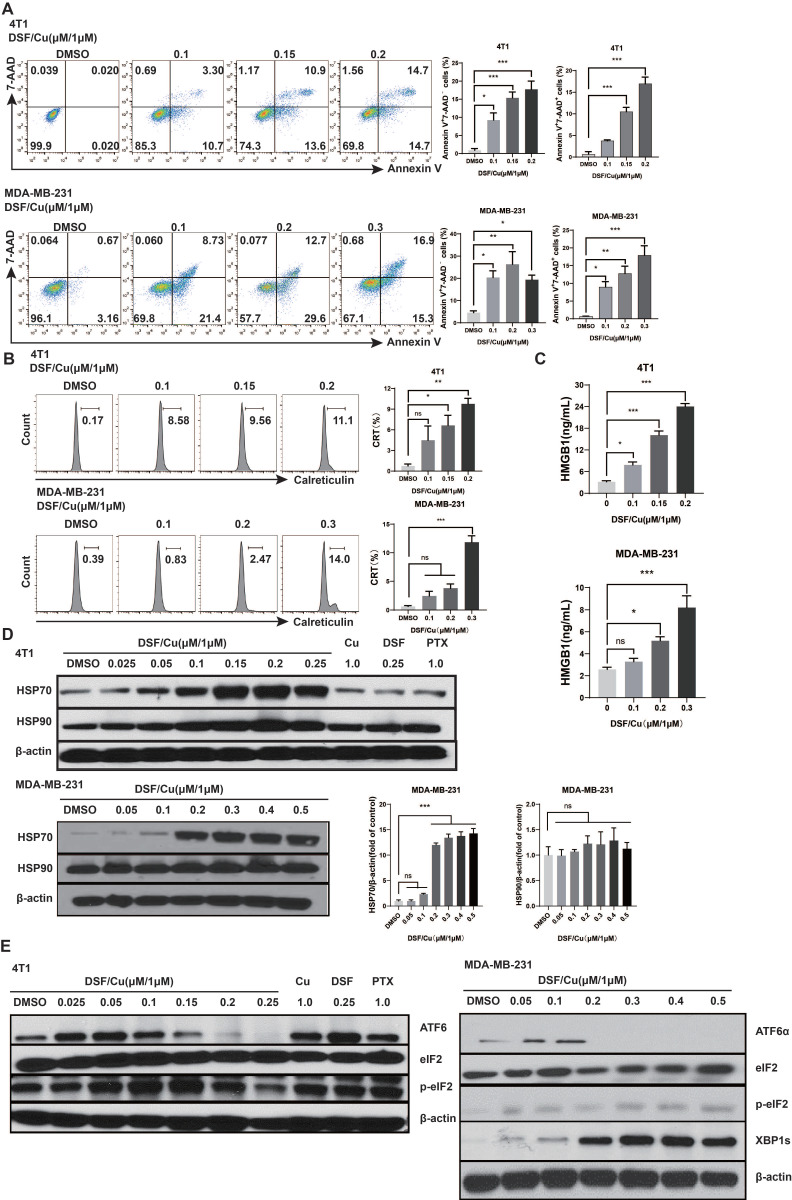
** DSF/Cu induced breast cancer cell death and DAMPs release.** Mouse 4T-1 and human MDA-MB-231 cells were treated with the indicated concentrations of DSF/Cu for 24h and then detected for apoptosis (7-AAD and Annexin V) (**A**) and the translocation of calreticulin (**B**) by flow cytometry. After 24 h of DSF/Cu treatment, extracellular HMGB1 protein levels were analyzed by ELISA (**C**), and cellular HSP70 and HSP90 protein levels were analyzed by western blot (**D**). DSF/Cu induced ER stress leading to ICD in breast cancer cells, as detected via the UPR branch downstream signaling proteins XBP1s, ATF6α and p-eIF2α** (E)**. Data are from three independently replicated experiments. All data were representative of mean±SEM. (*ns* no significance, *p < 0.05, **p < 0.01, ***p < 0.001 by Student's unpaired t-test.)

Triple-negative breast cancer (TNBC) is the most aggressive subtype of breast cancer. Therefore, we used the 4T1 and MDA-MB-231 cells *in vitro*. 4T1 and MDA-MB-231 cells were treated with different concentrations of DSF for 24 hours with a fixed 1 μM Cu^2+^. Flow cytometry analysis showed that after DSF/Cu treatment, the proportion of early and late apoptotic breast cancer cells increased compared with the control group (**Fig. 1A**). The contribution of DSF/Cu to ICD in breast cancer cells was further assessed by examining the exposure and release of DAMPs with immunogenic characteristics. The CALR is translocated to cell membrane surface when tumor cells undergo ICD^15^. Flow cytometry analysis revealed that DSF/Cu lead to an increase in surface CALR for 4T1 and MDA-MB-231 in a DSF dose-dependent manner (**Fig. 1B**). At the same time, the cell supernatant was collected and analyzed for HMGB1 by ELISA. The results showed that DSF/Cu increased the release of extracellular HMGB1 in a dose-dependent manner (**Fig. 1C**). The levels of heat shock proteins (HSP) 70 and HSP90 were detected by Western Blot. Compared with the control group, DSF/Cu does-dependently up-regulated the protein levels of HSP70 in MDA-MB-231 cells (p<0.001), while HSP90 protein showed no significant change (**Fig. 1D**). In addition, there are also significant up-regulation of HSP70 and HSP90 in 4T1 cells (**Fig. 1D**).

DSF converts to diethyldithiocarbamate (deDTC) within cells, two molecules of which bind one copper ion (Cu^2+^) to form the Cu [deDTC] complex (DSF/Cu)^16^. Published evidences have shown that DSF/Cu targets the p97 segregase adaptor NPL4^10^, inhibits NF-κB^17^, and activates endoplasmic reticulum stress by upregulating the inositol requiring-enzyme 1 alpha (IRE1α)-X-box-binding protein 1 (XBP1) axis, which leads to autophagic apoptosis^14^. We verified the activation of UPR-related pathway, consistent with our previous findings, DSF/Cu induced endoplasmic reticulum stress leading to ICD in BC cells through mainly activation of the XBP1 axis, rather than ATF6 or eIF2, all of which are downstream branches of the UPR (**Fig. 1E**)^9^.

### DSF/Cu activates endogenous type I IFN signaling and DCs maturation

**Figure 2:**
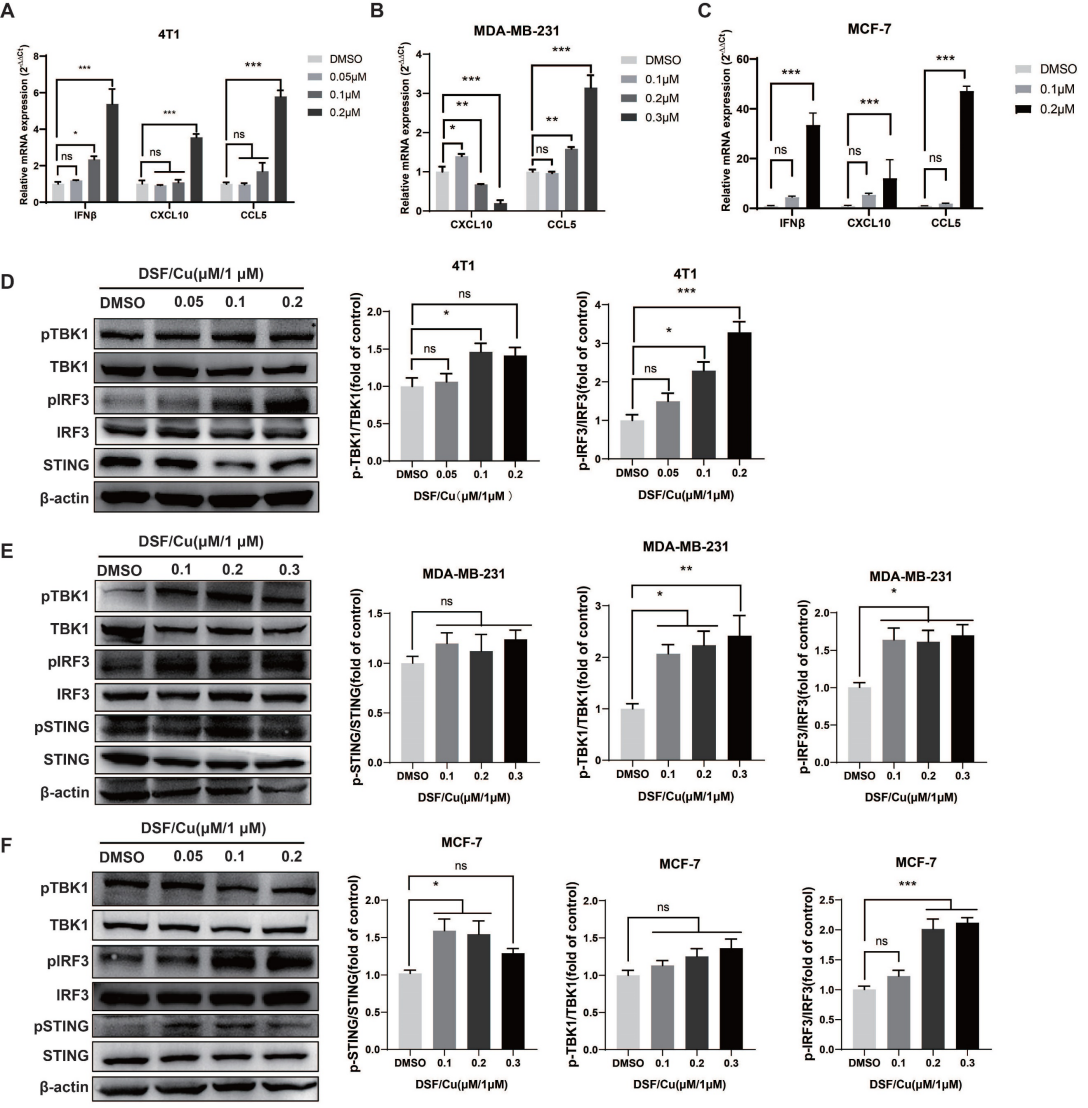
** DSF/Cu activated breast cancer cells endogenous cGAS-STING pathway.** *IFN-β*, *CXCL10* and *CCL5* mRNA expression were measured by qPCR in 4T1 (**A**), MDA-MB-231 (**B**) and MCF-7 (**C**) cells 24 h after DSF/Cu treatment. The protein levels of STING, pSTING, TBK1, pTBK1, IRF3 and pIRF3 in 4T1 (**D**), MDA-MB-231 (**E**) and MCF-7 (**F**) cells were measured by western blotting. Data are from three independently replicated experiments. All data was representative of mean±SEM. (*ns* no significance, *p < 0.05, **p < 0.01, ***p < 0.001 by Student's unpaired t-test.)

ICD-induced apoptotic tumor cell release DAMPs, therefore activate Toll‑like receptor 3 to promote type I IFN secretion and further produce CXCL10, which induces DCs maturation and stimulates T cell recruitment to enhance innate immunity^15^, ^18^. We further assessed the expression of type I IFN signaling-related cytokines in DSF/Cu-treated cells. The data showed that the mRNA expression levels of *IFN-β*, *CCL5,* and *CXCL10* were significantly up-regulated at increasing DSF/Cu concentrations in mouse 4T1 cells (**Fig. 2A**)**.** In human MDA-MB-231 cells, the mRNA expression of* CCL5* was significantly up-regulated after high concentration of DSF/Cu treatment, while the mRNA expression *CXCL10* was increased at low concentration (0.1μM) **(Fig. 2B)**. We also observed that the mRNA level of *IFN-β*, *CCL5,* and *CXCL10* were significantly up-regulated at increasing DSF/Cu concentrations in human MCF-7 cells **(Fig. 2C)**.

IRF3 is the main effector of the type I IFN signaling pathway. Western blot results showed that the levels of pIRF3 in the three breast cancer cells significantly up-regulated after DSF/Cu treatment, while the protein levels of pSTING and pTBK1 also showed an increasing trend **(Fig. 2D-F)**. Taken together, our data demonstrate that DSF/Cu activates endogenous type I IFN gene signaling in breast cancer cells, leading to cascade activation of antitμMor innate immunity.

**Figure 3:**
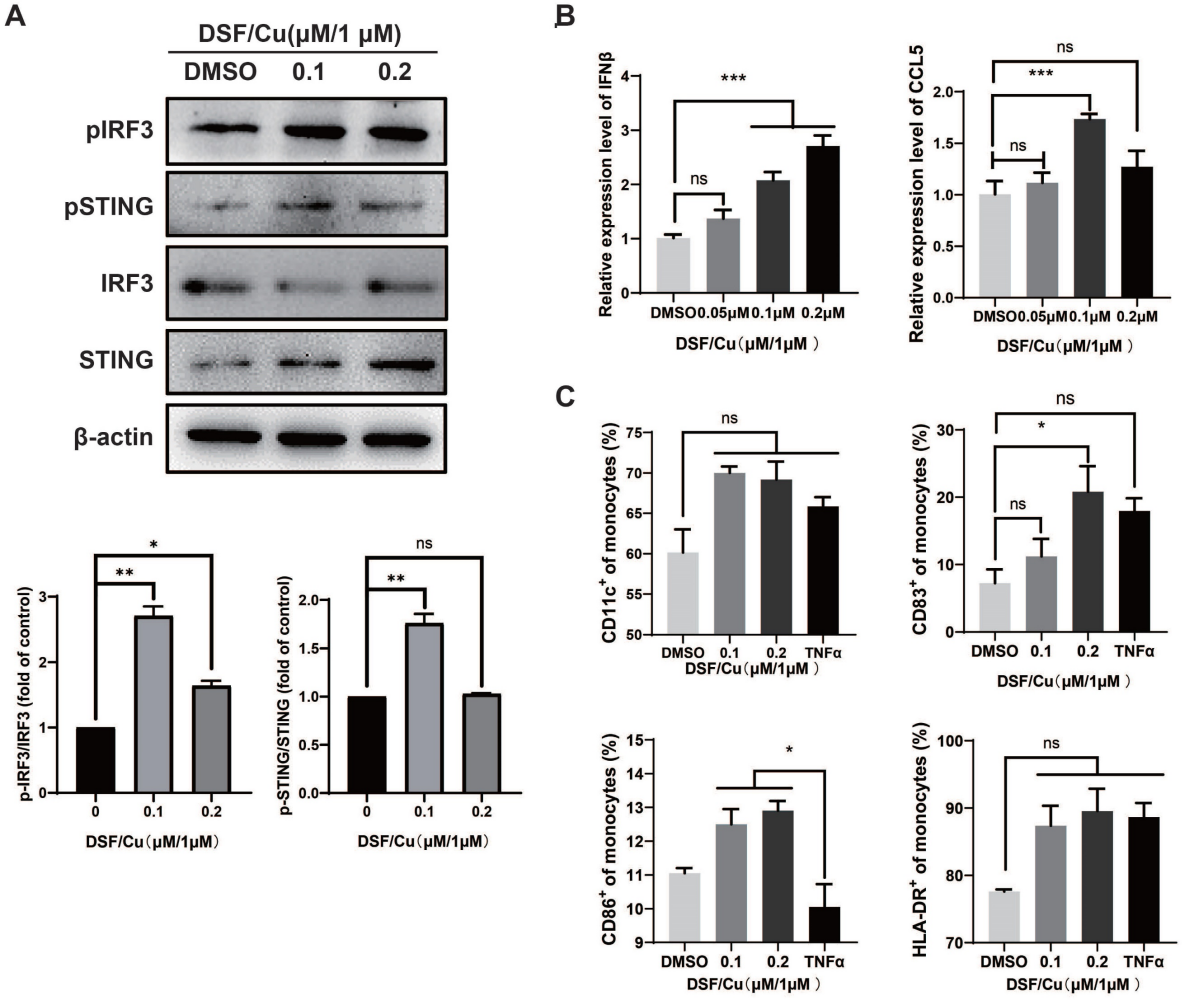
** DSF/Cu-treated breast cancer cells induced DCs maturation and activation of type I IFN pathway in DCs.** DCs were harvested 48h after co-culture with DSF/Cu-treated breast cancer cells. Western blotting of markers in the type I IFN pathway including total and phosphorylated (p) STING, and pIRF3 in lysates were analyzed from co-cultured DCs (**A**). *IFN-β* and *CCL5* mRNA expression of co-cultured DCs was measured by qPCR (**B**). The surface markers CD11c, HLA-DR, CD83, and CD86 of human monocyte-induced DCs were detected by flow cytometry (**C**). Data are from three independently replicated experiments. All data was representative of mean±SEM. (*ns* no significance, *p < 0.05, ***p < 0.001 by Student's unpaired t-test.)

Act as a bridge between adaptive immunity and innate immunity, Dendritic Cells (DCs) playing an important role in the uptake and presentation of tumor antigens, and further inducing the recruitment and activation of T cells 19. Human naïve mo-DCs from healthy donors were co-cultured with DSF/Cu-treated MCF-7 for 48 hours. We found that DSF/Cu also induced the activation of STING/IRF3 pathway **(Fig. 3A)** and increased the mRNA levels of *IFNβ* and *CCL5* in DCs **(Fig. 3B)**. Subsequently, TNFα-induction was used as a positive control to detect the activation of DCs. The results showed that CD83 expression was significantly up-regulated after DSF/Cu treatment, while HLA-DR and CD86 showed an up-regulated trend but not significantly different (**Fig. 3C**).

Based on the above results, it is speculated that DSF/Cu-induced DAMPs activates the expression of type I IFN and promotes DCs maturation by STING/IRF3 pathway.

### DSF/Cu enhanced the anti-tumor effects of in TNBC mouse models

**Figure 4:**
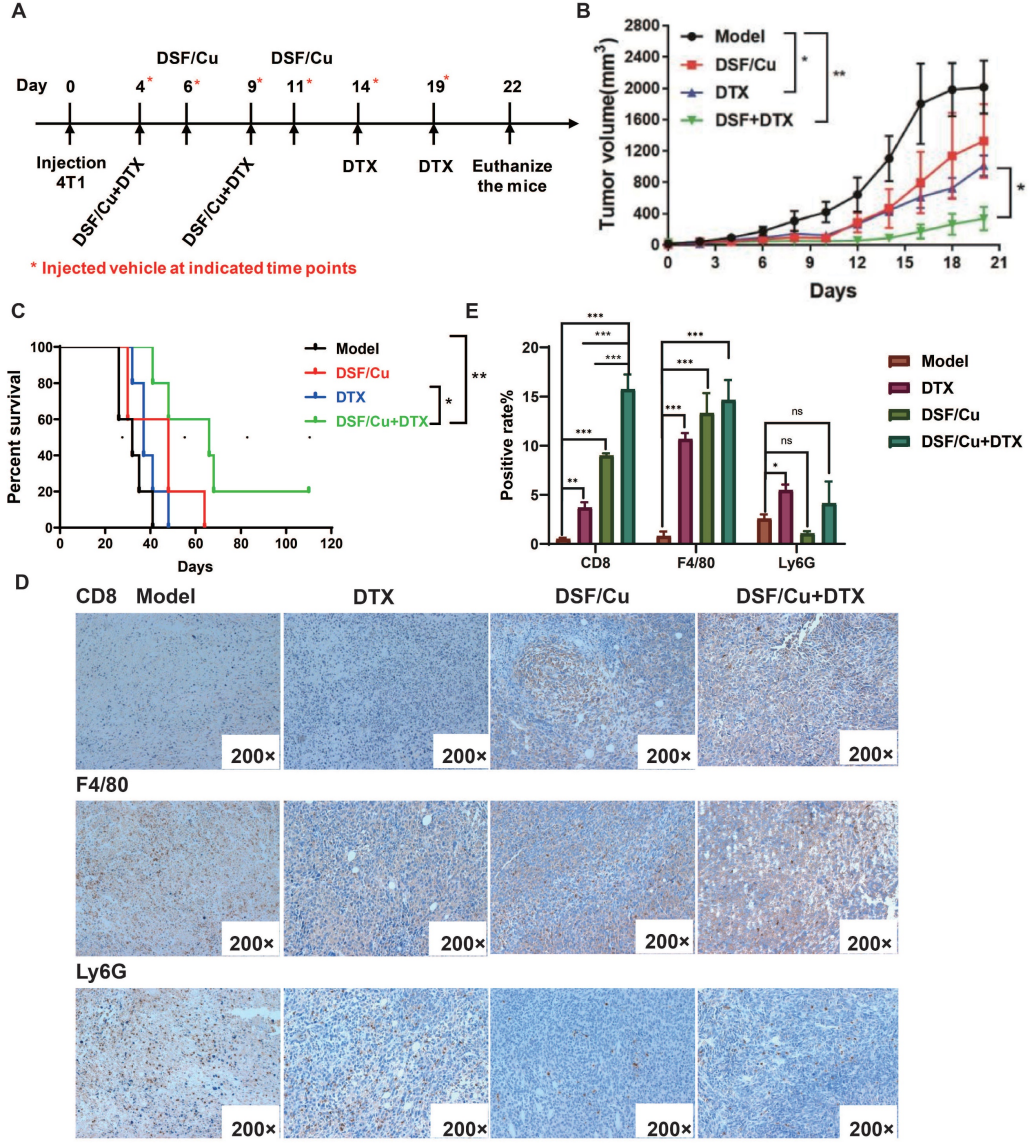
** DSF/Cu enhanced the anti-tumor effect of the chemotherapy drug DTX in immunocompetent mice.** (**A**) Schematic diagram of the establishment and dosing regimen of the 4T1 tumor-bearing mouse model. (**B**) Tumor volumes in 4T1-bearing mice were measured every two days after treatment with different regimens. (**C**) Percent survival of 4T1-bearing mice in different groups. (**D**) Immune infiltrates of tumors were analyzed by IHC for CD8, F4/80 and Ly6G expression after treatment with different regimens. The positive cells are stained yellow. (**E**) Positive rate for CD8+, F4/80+ and Ly6G+ cells infiltration. Data in B-E are from a single experiment representative of analysis performed in our groups with a total of 20 mice (n=5 for each group). All data representative of mean±SEM. (*ns* no significance, *p < 0.05, **p < 0.01, ***p < 0.001by Student's unpaired t-test.)

DTX-based combination chemotherapy is the most promising treatment option for breast cancer especially terminal or metastatic breast cancer^20^, but drug resistance is one of the most urgent issues to be addressed. Referring to the strategy of combining immunotherapy and chemotherapy in clinical research on TNBC, the combination of DSF/Cu and DTX is expected to enhance the immunogenicity of tumor cells through DSF/Cu, which can improve the effect of chemotherapy, reduce drug toxicity and tolerance. We established the TNBC mice model of in immunocompetent BALB/c mice. Based on tumor size, the female BABL/c mice were assigned into 4 groups(n=5) randomly on the 3rd day followed by a challenge with 4×10^5^ 4T1 cells. The tumor-bearing mice were injected intratumorally with 1.5 μM DSF and 1μM Cu each on the 4^th^, 6^th^, 9^th^ and 11^th^ day after molding, model group was injected with the vehicle on the 4^th^, 6^th^, 9^th^ ,11^th^,14^th^ and 19^th^, and DTX were injected intravenously every 5 days **(Fig. 4A)**. As shown in **Fig. 4B**, compared with the model group and the DTX group, the combination therapy significantly inhibited tumor formation, while DSF/Cu single had a slight inhibitory effect on tumor growth. Compared with the model group and the DTX group, the combined treatment significantly prolonged the survival of the mice, and the 4T1 tumor-bearing mice eliminated 20% (1/5) of the tumor, achieving long-term survival **(Fig. 4C)**.

The inflamed tumor microenvironment (TME) suggests positive prognostic for patients with advanced solid tumors 21. The phenotypes of an inflamed TME include tumor-infiltrating lymphocyte (TIL), proinflammatory cytokines, as well as macrophages and type I IFN signature. Decreased inflammatory phenotypes includes Foxp3^+^ T cell, MDSC and inflammatory cytokines^21^. To determine whether tumor growth inhibition was related to intratumoral infiltration of pro-inflammatory cells, CD8 F4/80 and Ly6G HIC staining were performed on the resected tumors **(Fig. 4D)**. The positive cells are shown yellow or brownish yellow, and negative cells are blue. For CD8^+^ cells, there is a significant increase in positive rate of combination therapy group compared with vehicle and monotherapy, which means a higher CD8^+^ cells infiltration (**Fig. 4E**). F4/80^+^ is the marker for mature macrophages. Similar with CD8+ cells, the tumor macrophages infiltration in combination group is also increased. Neutrophils promote the establishment of inflammatory microenvironment in the tumor and support tumor cell proliferation through multiple paracrine pathways. Therefore, the detection of neutrophil infiltration after treatment is also a very important indicator. **Fig. 4D, E** shows a similar neutrophils infiltration (Ly6G^+^ cells) of tumor tissue in combination therapy group.

**Figure 5:**
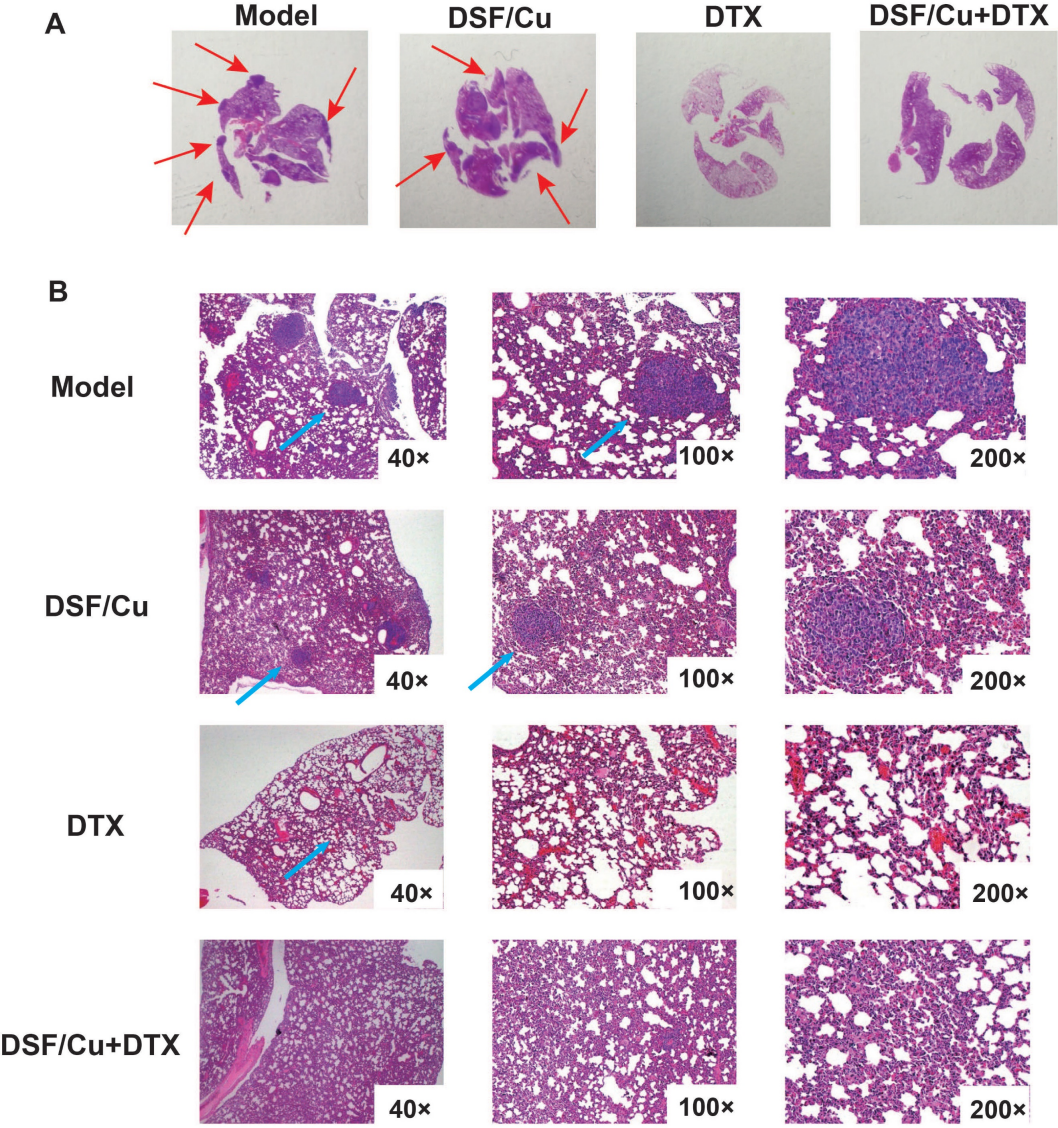
** DSF/Cu combined with DTX prevents lung metastasis in immunocompetent mice.** (**A**) Representative tumor nodules on the surfaces of lungs of 4T1-bearing mice. (**B**) Representative H&E of the lung sections from the model, DSF/Cu alone, DTX alone, and combination-treated group.Data in A-B are from a single experiment representative of analysis performed in our groups with a total of 20 mice (n=5 for each group).

Metastasis is the leading cause of death in breast cancer patients, and the lung is one of the most common organs for metastasis. We evaluated the pulmonary metastasis by hematoxylin and eosin (H&E) staining. The 5-year overall survival rate of patients with lung metastases is only 16.8%^22^. As shown in **Fig. 5A-B**, the lung surface sections of tumor-bearing mice in the model group and DSF/Cu group showed metastatic nodules, but the lung metastases in the DSF/Cu group were smaller than those in the model group. Observation under a high magnification microscope showed that compared with the model group, the treatment group showed less lung metastasis in mice. Especially in the DTX group and the combined treatment group, no lung metastatic nodules were observed, and the number of tumor cells infiltrated less and effectively reduced the occurrence of lung metastases in tumor-bearing mice.

Therefore, the combination therapy of DSF/Cu and DTX effectively inhibited 4T1 tumor growth, prolonged the survival time and reduced lung metastasis of tumor-bearing mice. This phenomenon might be related to the increase of CD8^+^ T cells and macrophages and decreased infiltration of MDSCs in tumor tissue.

### Combination therapy activates systemic immunity in TNBC model

**Figure 6:**
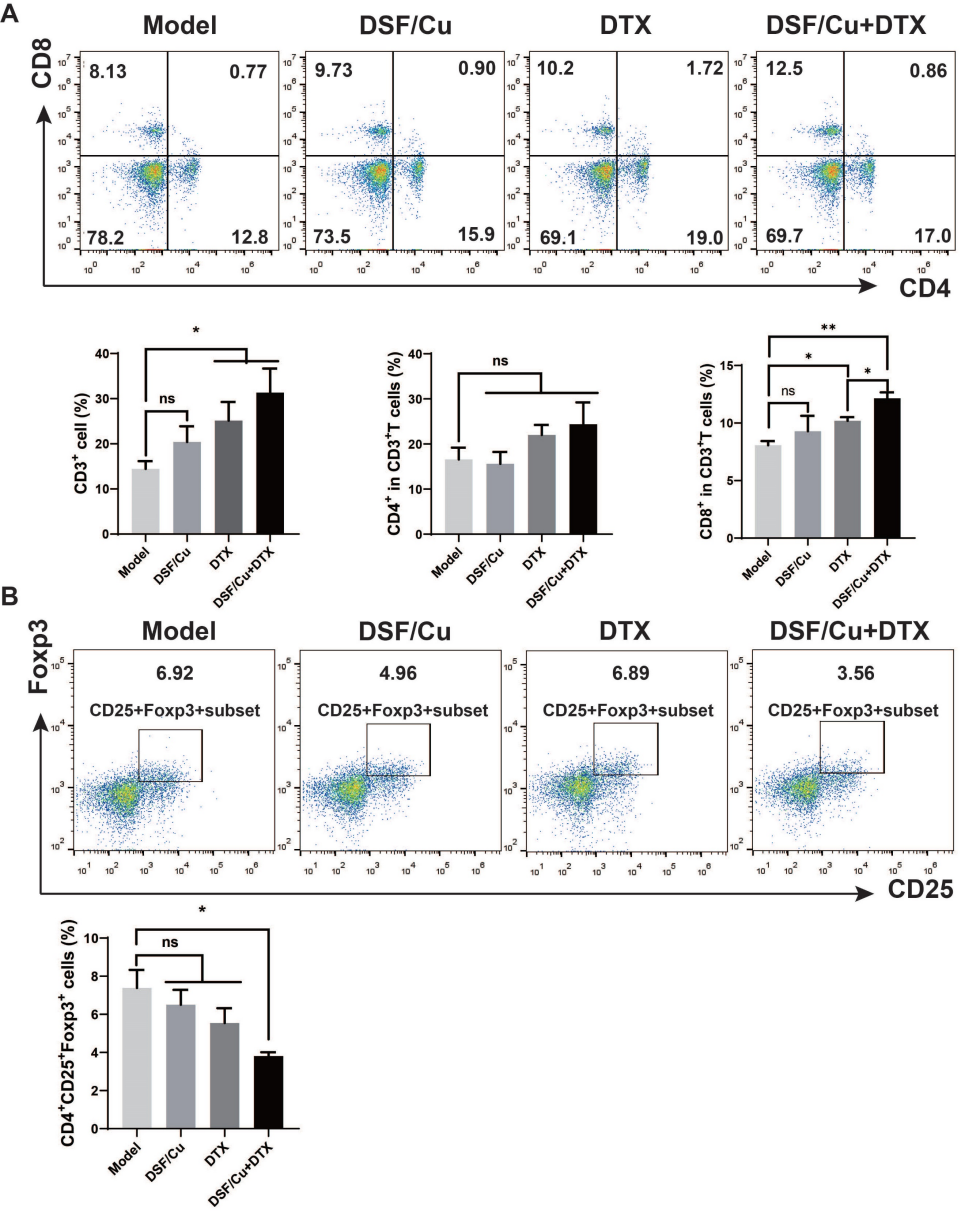
** Combination therapy induces a systemic immune response in 4T1-bearing mice.** Single-cell suspensions of spleens were analyzed for CD3^+^, CD3^+^CD4^+^, CD3^+^CD8^+^(**A**), and CD4^+^CD25^+^Foxp3^+^ T cells (**B**) by flow cytometry. Data in A-B are from a single experiment representative of analysis performed in four groups with a total of 20 mice (n=5 for each group). All data representative of mean±SEM. (*ns* no significant difference, *p < 0.05, ***p < 0.001 by Student's unpaired t-test.)

The long-term antitumor effect needs to be measured by the activation of systematic immunity. To further determine the immunomodulatory effect of the combination therapy, we then explored T cell activation in the spleen. Single-cell suspension of the spleen was obtained on day 30 for the detection of the proportion of immune cells by flow cytometry. Compared with the model group, the combined treatment of DSF/Cu and DTX increased CD3^+^ T cells in the spleen of tumor-bearing mice (p < 0.05), among which the proportion of CD8^+^ T cells was significantly increased (p < 0.001), and the number of CD4^+^ T cells increased with no statistical difference **(Fig. 6A)**. Regulatory T (Treg) cell is an immunosuppressive subset of CD4^+^ T cells. It play an important role in maintaining self-tolerance and immune homeostasis^23^. Treg cells have been shown to diminish the efficacy of many immunotherapies by suppressing the activity of CTL cells. As shown in **Fig. 6B**, compared with the model group, the combination treatment significantly reduced the proportion of CD4^+^CD25^+^Foxp3^+^ Treg cells (P<0.05).

## Discussion

According to the NCCN Guidelines, taxane-based chemotherapy is indicated for the treatment of metastasis patients. However, the main challenges of chemotherapy are cytotoxicity and drug resistance^24^, and immunosuppressive tumor microenvironment (TME) is one of the triggers^25^. Immunosuppressive TME will lead to drug resistance by inhibiting the proliferation of CD8+T cells, hindering drug absorption and inducing the paracrine growth factors to signal cancer cell growth^26^.

Through overcoming the immunosuppressive TME and drug- or mutation-induced systemic immune tolerance, immunotherapy has shown certain advantages in the treatment of breast cancer by activating the host's immune system to induce durable antitumor responses^27^,^28^. However, monotherapy shows limited efficacy, and clinical studies prefer to combine immunotherapy with standard chemotherapy^24^. For example, when Atezolizumab was initially used in a clinical study of PD-L1-positive patients in TNBC, the phase I results showed that the overall response rate (ORR) was only 10%^29^. The combination of Atezolizumab and nab-paclitaxel achieved promising results. A phase III clinical trial showed that the combination therapy increased median progression-free survival by 2.5 months compared with chemotherapy alone, and overall survival increased by 7 months^30^. Another marketed antibody, Pembrolizumab, was even more effective in combination than chemotherapy alone, with phase II/III results showing a three-fold higher pathological complete response (pCR) in patients with PD-L1-positive TNBC after combination chemotherapy^31^. However, the high cost of immunotherapy imposes a significant treatment burden on patients, which makes the clinical benefit remains limited.

Disulfide, an ancient alcohol drug with a good safety profile and low cost, is effective against multiple types of cancer in preclinical studies^6^,^32^. DSF complexed with Cu can produce the tumor cytotoxic metabolite^10^. Our study found that DSF/Cu treatment increases apoptosis in the breast cancer cells, and ICD-related indicators including CARL, HMGB1, HSP70, and HSP90 were upregulated. DSF/Cu-induced cell death confers immunogenicity on dying cancer cells, making it possible for dying cancer cells to act as endogenous vaccines that enhance immune responses and induce long-lasting antitumor activity. Induction of ICD in tumor cells may trigger the release of mt DNA or RNA, which is essential for the activation of type I interferon signaling^33^,^34^. IFN-β can recruit and activate CD8^+^ DCs to promote cross-presentation with antigen-specific CD8^+^ T cells^35^. Therefore, the expression of type I interferon may be essential for anti-tumour immunity. We found that DSF/Cu up-regulated the expression of CXCL10, CCL5 and IFN-β in multiple high metastatic breast cancer cell lines, which was associated with the activation of the STING-TBK1-IRF3 pathway. We further simulated the effect of ICD on DCs in vitro by co-incubating DSF/ Cu-treated breast cancer cell supernatant with immature mo-DC. Similarly, in human primary DCs we also observed the activation of STING-TBK1-IRF3 pathway and the up-regulation of CCL5 and IFN-β. The high concentration of DSF (0.2 μM) showed the decrease of pIRF3 and pSTING compared with DSF (0.1 μM). We speculate that DSF at 0.2 μM causes more cell death to result in less cells for the detection. The IC50 values of DSF in 4T1, MDA-MB-231 and MCF-7 cells were 0.126μM、0.380 μM and 0.227 μM in our previous work^36^, all close to 0.2 μM. However, the actual reason for the decrease of pIRF3 and pSTING by the high concentration of DSF is still not clear, and needs further investigation. And after 48h, the maturation of DCs was detected. These results suggest that DSF/Cu may be able to induce DCs maturation by stimulating the activation of type I interferon-related pathways in tumour cells. The elucidation of this mechanism is important for understanding the anti-tumour effects of DSF/Cu and provides new evidence for the role of DSF/Cu in the regulation of intrinsic and adaptive immunity.

Owing to its high invasiveness, TNBC mice model were used to test the effect of the combined strategy between DSF/Cu and DTX Based. DSF/Cu can theoretically compensate for the tolerance problem of DTX in breast cancer, and exert stronger anti-tumor effects. The results show that the combination therapy can effectively inhibit tumor growth, prolong the survival period and inhibit lung metastases of 4T1 tumor-bearing mice. Major mechanisms of tumor immune escape include the downregulation of antigen processing and presentation components, recruitment of suppressive immune cells, such as regulatory T cells, myeloid-derived suppressor cells (MDSCs), and tumor-associated macrophages, production of soluble factors associated with immunosuppression such as TGF-β1 and IL-10^37^. Our previous study showed that the combination of DSF/Cu and DTX increased the infiltration of CD8^+^ T cells and macrophages in tumor tissue, decreased the infiltration of MDSCs, and increased the activation of CD8^+^ and CD4^+^ T lymphocytes in the spleen of mice. Meanwhile, significantly reduced the level of Treg cells, which are critical for disrupting tumor immune evasion^37^. Therefore, these findings suggest that the enhanced antitumor effect of DTX by DSF/Cu is associated with the alteration in TME. We propose that dying tumor cells killed by combination therapy may act as in situ tumor cell-specific vaccines to activate T-cell responses in mice and generate inflammatory TME with profound "ectopic effects".

In conclusion, DSF/Cu was verified as an ICD inducer to induce apoptosis and the release of DAMPs in multiple breast cancer cells and further promoted DC maturation by activating the type I interferon signaling pathway. DSF/Cu concurrent with DTX significantly retarded tumor progression and induced systemic immune response. Unlike previous studies, this study presents a new therapeutic approach by expanding the scope of typical chemotherapeutic drug for metastasis breast cancer, that is, DSF/Cu or other ICD inducers can be used as an adjuvant to conventional chemotherapy for long-term efficacy. In addition, we demonstrated the adaptive immune responses induced by DSF/Cu, which is the foundation for combinational therapy to achieve more effective therapeutic effects and less adverse reactions. The antitumor spectrum and better binding agent of DSF/Cu need further study to achieve the best therapeutic effect.

## Figures and Tables

**Table 1:** qPCR primer sequences

Primer name	Forward primer (5'-3')	Reverse primer (5'-3')
Mouse CXCL10	CCAAGTGCTGCCGTCATTTTC	TCCCTATGGCCCTCATTCTCA
Mouse IFNβ	ATGAACTCCACCAGCAGACA	ATCCAGGCGTAGCTGTTGTA
Mouse CCL5	GCTGCTTTGCCTACCTCTCC	TCGAGTGACAAACACGACTGC
Mouse β-actin	GTGACGTTGACATCCGTAAAGA	GCCGGACTCATCGTACTCC
Human IFN-β	CAGCAGTTCCAGAAGGAGGA	AGCCAGGAGGTTCTCAACAA
Human CCL5	CCAGCAGTCGTCTTTGTCAC	CTCTGGGTTGGCACACACTT
Human CXCL10	TGGATGTTCTGACCCTGCTT	GGCAGTGGAAGTCCATGAAG
Human β-actin	CATGTACGTTGCTATCCAGGC	CATGTACGTTGCTATCCAGGC
